# C2c: turning cancer into chronic disease

**DOI:** 10.1186/gm555

**Published:** 2014-05-28

**Authors:** Stephan Beck, Tony Ng

**Affiliations:** 1 Medical Genomics, Cancer Biology Department, UCL Cancer Institute, Paul O’Gorman Building, University College London, London WC1E 6BT, UK; 2 Richard Dimbleby Department of Cancer Research, Randall Division and Division of Cancer Studies, Kings College London, Guy’s Medical School Campus, London SE1 1UL, UK; 3 Department of Molecular Oncology, UCL Cancer Institute, Paul O’Gorman Building, University College London, London WC1E 6BT, UK; 4 Breakthrough Breast Cancer Research Unit, Department of Research Oncology, Guy’s Hospital King’s College London School of Medicine, London SE1 9RT, UK

## 

Despite fantastic progress in research over the past decade, cancer remains a major source of mortality worldwide. In the UK, for instance, cancer overtook circulatory diseases as the leading cause of death in 2011 [1]. Early detection would obviously be best to reduce this burden, but it requires exquisitely sensitive technology and population-wide screening programs. Short of finding a cure for cancer, turning cancer into a clinically manageable chronic disease like diabetes would be a major step forward. In this Editorial, we introduce a *Genome Medicine* series on cancer epigenomics [http://genomemedicine.com/series/cancerepigenomics], and discuss progress towards turning cancer into chronic disease with a focus on epigenomics.

For cancers for which appropriate treatment options are available, the proposed cancer to chronic disease (C2c) approach (Figure 1) requires two key components: first, knowledge of the precise localization and quantification of the cancer burden anywhere in the body, which can be achieved by non-invasive whole-body imaging [2,3]; and second, knowledge of the precise molecular signature of the evolving cancer burden to reiteratively tailor treatments predicted to be most effective in combating the establishment of resistance and subsequent relapse. This can be achieved by (epi)genomic profiling of cancer-specific components isolated from minimally invasive blood samples, also known as liquid biopsies [4]. After all, ‘Blut ist ein ganz besonderer Saft,’ as already noted by Faust - the scholar who was striving to know everything - in Johann Wolfgang von Goethe’s 1808 play *Faust Part I*[5].

**Figure 1:**
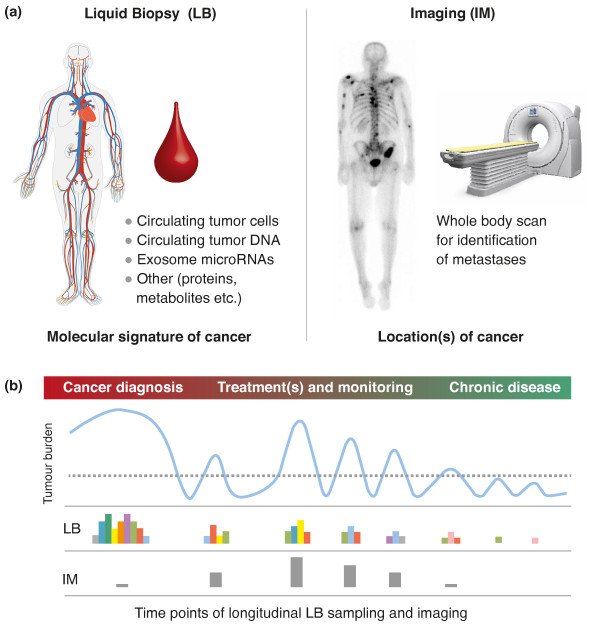
**Illustration of the cancer to chronic disease (C2c) approach. (a)** C2c core technologies: liquid biopsies (LB) and whole-body imaging (IM). For LB, current assays include circulating tumor cells and circulating tumor DNA as well as microRNAs. Examples of other biomarkers are prostate-specific antigen as exemplar protein biomarker, and 2-hydroxyglutarate as exemplar epigenetic oncometabolite, which is a potent inhibitor of TET demethylases. For IM, current platforms include, for example, magnetic resonance imaging, positron emission tomography and sodium iodide symporter imaging. Images: circulatory system image obtained from Wikimedia Commons (LadyofHats); magnetic resonance image of prostate cancer metastases obtained from Wikimedia Commons (Radswiki); whole body scanner image obtained with permission from Hitachi Medical Systems. **(b)** Example of longitudinally measured LB and IM signals in response to treatment. The color code in the LB track represents the evolving omic heterogeneity of the cancer, which in turn informs the next best treatment(s). The dotted line denotes the threshold between cancer and chronic disease. The aim is to keep both the LB and IM signals within the range defined as chronic disease.

## The (epi)genomic landscape of cancer

Cancer is essentially a disease of the genome and the epigenome. To understand the (epi)genomic landscape of cancer, therefore, requires comprehensive analysis of both mutations and epimutations, which is exactly what the Cancer Genome Atlas [http://cancergenome.nih.gov/], the International Cancer Genome Consortium [http://www.icgc.org] and related efforts such as BLUEPRINT [http://www.blueprint-epigenome.eu/] are in the process of doing for all major types of cancer. The first pan-cancer analyses of the data generated so far have already revealed valuable insights into commonalities, differences and emergent themes across tumor lineages with regard to mutations [6,7] and somatic copy number alterations [8]. Similar analyses are now required for epimutations as well as combined and combinatorial effects of genomic and epigenomic alterations. The recent discovery of numerous and highly recurrent mutations in epigenome modifiers such as specific DNA methyltransferases (for example, *DNMT1*) and histones (for example, *H3.3*) have highlighted the importance of epigenetic mechanisms in cancer [9]. Indeed, in many cancers, epimutations occur more frequently than mutations [10]. The challenge for approaches such as C2c is to extract the key information for each cancer from the vastness of available data. This can be achieved by computational modeling to reduce complex multi-dimensional data into less complex biomarkers that are more suitable for downstream patient monitoring. A pioneering example of this is OncoTrack [http://www.oncotrack.eu/], Europe’s largest public-private biomarker consortium, in which this approach is applied to colorectal cancer.

## Liquid biopsy and imaging - the bow and arrow of C2c

The appeal of using liquid versus tumor biopsies for patient monitoring is obvious and well documented [3]. First, liquid biopsies allow longitudinal sampling using a routine and minimally invasive procedure (blood sampling). Second, they have the potential to capture the majority of the cancer burden and not just the primary tumor or metastases that are accessible through solid biopsies. Third, liquid biopsies are information-rich.

As illustrated in Figure 1, key components of the evolving cancer burden can be analyzed from a single liquid biopsy, such as circulating tumor cells shed from primary tumor and/or metastases, circulating tumor DNA isolated from blood plasma or serum, and cancer-specific microRNAs that are enriched in exosomes (50 to 200 nm vesicles), as well as proteins and onco-metabolites. Recent progress in digital PCR and targeted next-generation sequencing has revealed that circulating tumor DNA is detectable in the majority of cancer patients [11] and is informative for monitoring acquired resistance to cancer therapy [12].

In addition to localizing tumors as well as metastases, whole-body molecular imaging can also be used to non-invasively probe tumor heterogeneity, which describes the existence of subpopulations of cancer cells with distinct (epi) genotypic, proteomic and phenotypic variations. Specifically, it can provide important spatiotemporal information concerning tumor progression to aid treatment decisions for individual cancer patients. As examples, ErbB2/HER2 and sodium iodide symporter (NIS) imaging can be employed to discern the difference or discordance in protein expression (HER2 status, which is used in the clinic to assign therapies such as trastuzumab [13]) and the differential sensitivity to chemotherapy [14], respectively, between primary tumor and the corresponding metastases. For instance, patients who had a HER2-negative primary tumor may have HER2-positive metastases that may not be amenable to biopsy, so treatment such as trastuzumab may be incorrectly withheld if the clinical decision is based on the HER2 status of the archived primary tumor sample alone. Similarly, using preclinical NIS imaging, response to chemotherapy has been shown to be heterogeneous among the primary tumor and metastases in different organ/tissue microenvironments within the same animal. Molecular imaging may also provide a non-invasive means of monitoring and quantifying the emergence of potential treatment resistance mechanisms such as *ERBB2*[15] and *MET*[16] amplifications as well as *KRAS* mutations [17], which have been known to arise in response to selection pressure of targeted therapies [18]; the latter (treatment targeting EGFR, for example) have, to date, only achieved modest improvements in clinical outcome [19,20].

A key advantage of integrating liquid biopsy-based omics and imaging is to harness the combined sensitivities and specificities of molecular imaging and next-generation sequencing techniques in order to facilitate early detection of the treatment-resistant variants that evolve as a mechanism of acquired resistance. As the sensitivity of next-generation sequencing improves towards single cell resolution, molecular imaging would still provide complementary information that reports on the spatial heterogeneity of treatment response, such as between different metastatic sites [14], as well as elucidating the functional significance of the genomic changes observed in the plasma (for example, metabolic imaging in the context of *IDH1* and *IDH2* mutations [21]). For C2c, the combined use of imaging and liquid biopsies will provide the most comprehensive monitoring of the cancer burden that can be achieved by non-invasive technology.

## Bottlenecks

Despite many advances and the announcement of the US$1,000 genome earlier this year, technology remains a major challenge for analyzing liquid biopsies. In particular, epigenomic analyses need to catch up with what is already possible at the genomic level as mentioned above. For C2c to succeed in turning cancer into chronic disease, we would need to clinically adopt an effective surveillance strategy, namely, non- or minimally invasive monitoring of patients for treatment-resistant tumor variants that evolve as a mechanism of acquired resistance, as diabetes is chronically managed by monitoring blood glucose levels. An example of this would be chronic myelogenous leukemia (CML), a disease in which most patients still harbor residual disease despite an early identification of a specific chromosomal abnormality that can be targeted with a tyrosine kinase inhibitor (imatinib) [22]. An active surveillance program (combining molecular imaging - for example, Abl kinase positron emission tomography (PET) imaging using a radiolabeled small molecule inhibitor [23] - and liquid biopsies) could be used to track the molecular evolution of the Abl kinase that is subjected to treatment pressure, and where necessary, new second- and third-generation tyrosine kinase inhibitors can be introduced. Although several targeted therapies are available (for example, against EGFR, HER2/neu, HER3, BCR-ABL, PI3 kinase, Akt, MEK, BRAF, CD20, TOR and VEGF), more are needed, particularly for some cancers, such as pancreatic carcinoma, which is still associated with a dismal outcome and for which there are limited therapeutic options. Epigenetics may help to overcome this limitation by sensitizing cancers to existing treatments to which the cancer was not previously responsive. For example, recent pioneering studies have shown that treatment of cancer cell lines and patients with drugs targeting epigenetic alterations, such as DNA methylation, showed upregulation of immune-modulatory pathways, thus sensitizing these cancers for possible treatment with existing immune therapies [24,25].

## Outlook

There is currently unprecedented consensus among researchers, clinicians, politicians and the wider public that omics in one form or another will transform future healthcare, including the treatment and management of cancer. By combining omics with imaging, the C2c strategy introduced here will be one step towards this transformation. Once fully developed and established, it may also be applicable to early detection using population-wide screening and thus become an integral part of personalized medicine. Although much remains to be done, the progress made so far suggests that for C2c to succeed will not require ‘a pact with the devil’, unlike the case of poor Dr Faust who had to give his soul to Mephistopheles to succeed in his quest for ultimate knowledge.

## Competing interests

The authors declare that they have no competing interests.
